# Research progress and future directions on intraductal papillary mucinous neoplasm: A bibliometric and visualized analysis of over 30 years of research

**DOI:** 10.1097/MD.0000000000033568

**Published:** 2023-04-14

**Authors:** Jae Keun Park, Ji Woong Hwang

**Affiliations:** a Department of Internal Medicine, Kangnam Sacred Heart Hospital, Hallym University College of Medicine, Seoul, Republic of Korea; b Department of Surgery, Chung-Ang University Gwangmyeong Hospital, Chung-Ang University College of Medicine, Gwangmyeong, Republic of Korea.

**Keywords:** bibliometric analysis, intraductal papillary mucinous neoplasm, IPMN, keyword analysis, research focus, research trend

## Abstract

Malignant change from low-grade dysplasia to high-grade dysplasia and invasive carcinoma following an adenoma-carcinoma sequence is becoming more common in intraductal papillary mucinous neoplasm (IPMN) of the pancreas. The aim of this study is to analyze their main characteristics and recent research trends in IPMNs and consequently create better understandings of the current situation and trends.

**Methods:**

A comprehensive search was performed in The Science Citation Index Expanded of the Web of Science. All articles between 1990 and 2021 were searched. VOS viewer (Leiden University, Leiden, Netherlands) was used for a qualitative and quantitative analysis of keywords, constituting maps based on co-occurrence matrix.

**Results:**

A total of 1658 eligible articles were screened among the 3950 identified articles for this subject. Finally, 879 articles were included in this study. Many articles on IPMN have been published in Japan and South Korea. Tanaka published the highest number of articles (n = 26, citations = 11,143). The *Pancreas* published the highest number of articles. (n = 100, citations = 2533). These articles were grouped into 4 clusters including basic research, disease overview, management/prognosis and malignant IPMN by using bibliometric keywords network analysis. Overlay visualization demonstrates, a trend of the studies has been changed from basic research or disease to management or prognosis.

**Conclusions:**

In this study, we found and highlight the most cited and influential articles related to IPMN. Plus, this study analyzed global research trends in IPMN over the past 30 years and provides insight into the features and research hotspots of the articles in IPMN research.

## 1. Introduction

Intraductal papillary mucinous neoplasms (IPMN) of the pancreas which originated from the pancreatic ductal system has been considered a precursor lesion of malignant transformation.^[1–4]^ Morphologically, IPMN can be classified as main duct IPMN, branch duct IPMN (BD-IPMN) and mixed-type IPMN.^[5]^ Three types of IPMN represent a different potential of malignant transformation. The risk of malignant transformation of main duct IPMN and mixed-type IPMN has been reported from 35% to 100%. With BD-IPMN, the risk of malignant transformation has been reported to be between 6 and 51%.^[6]^ Plus, there are 4 types of IPMNs, which are classified based on histologic features: gastric, intestinal, pancreaticobiliary or oncocytic subtypes. Gastric-type IPMNs have the best prognosis, a 5-year survival rate of > 90%. Intestinal-type IPMNs are less favorable, with 5-year survival rates of 70%. The 5-year survival rates of pancreatobiliary-type IPMNs are 50%. Oncocytic IPMNs with associated cancer have a 5-year of 60%.^[7]^ The international consensus guidelines suggesting high-risk stigmata and worrisome features to determine the potential risk of malignant transformation of IPMN were established in 2006 and revised in 2017.^[3,5]^ Numerous articles have been published regarding IPMN in various fields, such as the molecular aspects, clinical presentation, diagnosis, and management.

Numerous bibliometric assessments examining the research performance of authors, countries, journals, and institutions, as well as the trends of publications with similar topics on a variety of topics, have been reported in recent years.^[8]^ Bibliometric analysis has become a popularly accepted approach to present the status and research pattern. Consequently, it is a crucial instrument for rapidly acquiring scientific information and evaluating the essential areas of research and emerging possible trends for future studies. Therefore, a bibliometric analysis is urgently needed to provide a comprehensive knowledge of IPMN and future research direction. However, there are only a few bibliometric studies published by using citation rank analysis in IPMN.^[9,10]^

In the present study, we analyzed the IPMN-related articles collected from the web of science (WOS) Core database in order to assess the available literature in terms of authors, year, journals, countries, institutions, keywords, and references. The purpose of this study was to use bibliometric methods to analyze recent 30-year trends in research in IPMNs and consequently create a better understanding of the current situation and trends of those research studies by assessing their main characteristics.

## 2. Methods

The Institutional Review Board approved the review of medical articles using a publicly available database (Kangnam Sacred Heart Hospital, Hallym University College of Medicine, Institutional Review Board No. NON2022-003). The study was conducted according to the Preferred Reporting Items for Systematic Reviews and Meta-Analyses guidelines.^[11]^

### 2.1. Data sources and search strategy

A comprehensive search was performed in The Science Citation Index Expanded of the WOS for bibliometric analysis. All articles between 1990 And 2020 were searched from WOS on October 25, 2021. Search strategy was as follows, TS = (“IPMN” OR “intraductal papillary mucinous neoplasm”).

### 2.2. Inclusion and exclusion criteria

Only original articles and reviews were included. Articles not written in English were excluded in this study. Among the articles obtained from WOS, the articles studied on biliary IPMN or mucinous cystic neoplasm or pancreatic cystic neoplasm or simple pancreatic cysts were excluded. Two independent authors reviewed the title and abstract of each article, and selected articles related to this study.

### 2.3. Data extraction and analysis

The included articles were analyzed using following parameters: publication year, country of origin, citation number, institutional affiliation, corresponding author’s name, publishing journal, topic, and keywords of articles. The Excel 365 (Microsoft Corp.) was used for data analysis and processing.

For classification of the topic of the articles, keyword analysis was performed using VOS viewer. VOS viewer divided the entire articles into 4 clusters as follows: basic research, disease overview, “management and prognosis,” and malignant IPMN. Five categories were finally determined after the topic “management and prognosis” was divided into management and prognosis, respectively, for detailed analysis, as follows: basic research, disease overview (including diagnosis, clinicopathological features, radiologic examination), management, prognosis (including natural history, postoperative follow-up, and recurrence), and malignant IPMN (including prediction and determination of malignancy, and malignant potential).

VOS viewer (Leiden University, Leiden, Netherlands) was used for a qualitative and quantitative analysis of keywords, constituting maps based on co-occurrence matrix.^[12]^ Keywords were represented in nodes in the map. A larger node indicates higher frequency, and lines between the nodes indicate the co-occurrence of specific keywords. Each cluster of relevant keywords is identified by a color. Overlay visualization represents developments over time by demonstrating the network map of the trend topics according to the keywords. The keywords in articles were manually standardized by authors, as different representations of the same keywords could result in errors.^[13]^

## 3. Results

**Figure 1.**
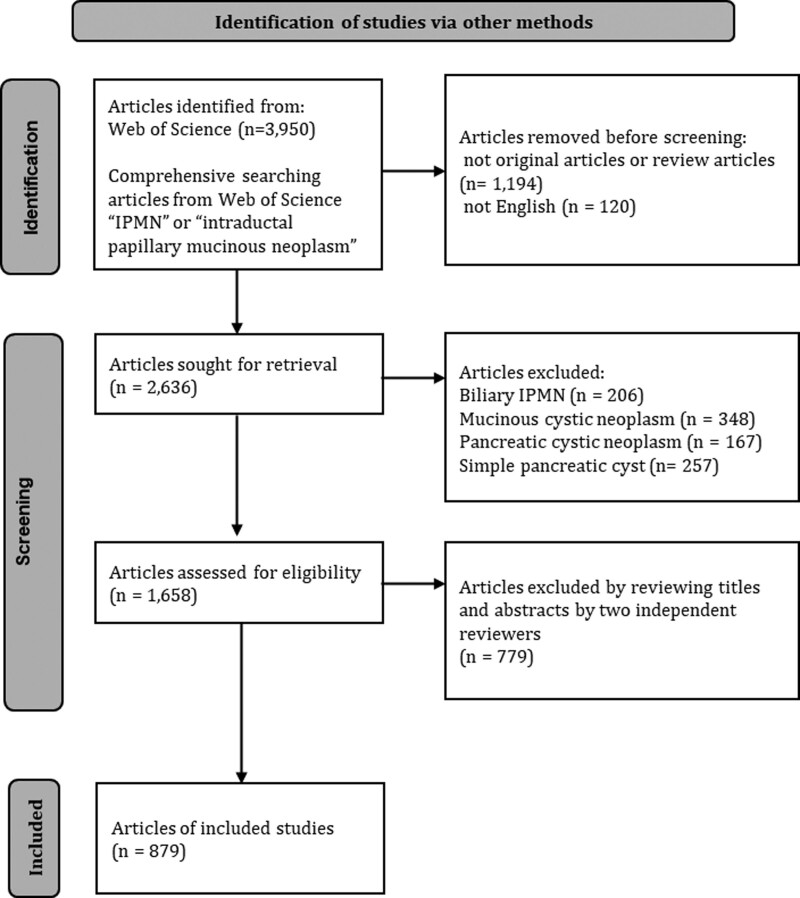
Flow diagram showing the selection for articles on intraductal papillary mucinous neoplasm.

A total of 1658 eligible articles were screened among the 3950 identified article for this subject. Finally, 879 articles were included in this study (Fig. 1). The top 10 most cited articles on IPMN are listed in Table 1. The most cited article was “Intraductal papillary mucinous neoplasms of the pancreas–An updated experience” by Yeo et al (634 citations). The extent of the increase in publication articles on IPMN study had been increasing until 2010 but has declined slightly since 2011 (Figure S1, Supplemental Digital Content, http://links.lww.com/MD/I814).

**Table 1:** Top 10 most cited articles on IPMN research.

Corresponding author	Country	Title	Published year	Total citations
Yeo, CJ	USA	Intraductal papillary mucinous neoplasms of the pancreas–An updated experience	2004	634
Furukawa, T	Japan	Classification of types of intraductal papillary mucinous neoplasm of the pancreas: a consensus study	2005	450
Chari, ST	USA	Study of recurrence after surgical resection of intraductal papillary mucinous neoplasm of the pancreas	2002	351
Sugiyama, M	Japan	Predictive factors for malignancy in intraductal papillary mucinous tumors of the pancreas	2003	326
Adsay, NV	USA	Pathologically and biologically distinct types of epithelium in intraductal papillary mucinous neoplasms–Delineation of an “Intestinal” pathway of carcinogenesis in the pancreas	2004	325
Castillo, CFD	USA	Branch duct intraductal papillary mucinous neoplasms: Observations in 145 patients who underwent resection	2007	317
Terris, B	France	Intraductal papillary mucinous tumors of the pancreas confined to secondary ducts show less aggressive pathologic features as compared with those involving the main pancreatic duct	2000	299
Schmidt, CM	USA	Intraductal papillary mucinous neoplasms–Predictors of malignant and invasive pathology	2007	297
Brennan, MF	USA	Intraductal papillary mucinous neoplasms of the pancreas–An analysis of clinicopathologic features and outcome	2004	264
Yeo, CJ	USA	Intraductal papillary mucinous neoplasms of the pancreas: An increasingly recognized clinicopathologic entity	2001	254

IPMN = intraductal papillary mucinous neoplasms.

### 3.1. Publication author, institution, and country

**Figure 2.**
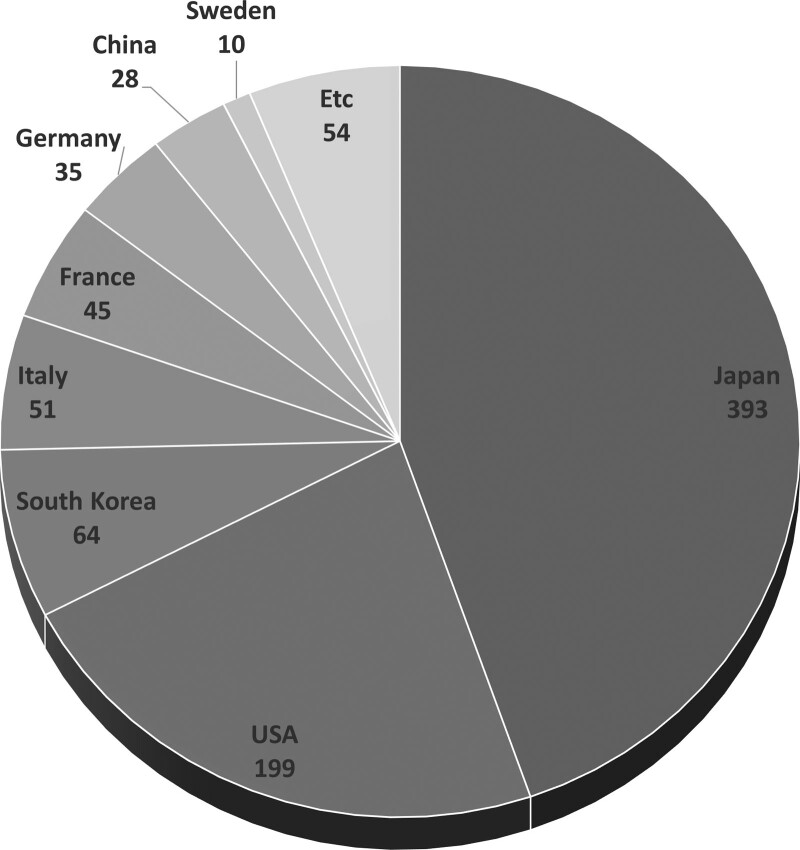
The contribution of country to publication on intraductal papillary mucinous neoplasm.

Japan (n = 393, 44.7%), which published less than half of the publications, was the most productive country (Fig. 2). The USA (n = 199, 22.6%) and South Korea (n = 64, 7.3%) followed. The order of publishing institutions shows the same trend as the order of publication country. In terms of international collaboration, it appears that the USA and Japan are leading the multicenter studies, and it is evident that several European and Asian nations are collaborating (Figure S2, Supplemental Digital Content, http://links.lww.com/MD/I815). The institution publishing the most articles was the Kyushu University (n = 54), followed by Seoul National University (n = 25), and the Memorial Sloan-Kettering Cancer Center (n = 18) (Table 2). According to institutions, there are numerous international collaborations between the USA, Japan, and Korea (Figure S3, Supplemental Digital Content, http://links.lww.com/MD/I816). Unlike bibliometric analyses of other diseases, many articles on IPMN have been published in East Asia countries such as Japan or South Korea. Tanaka published the highest number of articles and subsequently had the highest number of total citations (n = 26, citations = 1,1143). Following Tanaka, Jang JY (n = 13, citations = 318) and Schmidt CM (n = 12, citations = 666) each published the 2nd and 3rd the highest number of articles and total citations (Table 3).

**Table 2:** Top 10 institutions with highest articles production in IPMN research.

Rank	Institution	Country	Number of studies	Total citations	Average citation
1	Kyushu University	Japan	54	1916	35.5
2	Seoul National University	South Korea	29	1114	38.4
3	Johns Hopkins University	USA	21	2058	98
4	Memorial Sloan-Kettering Cancer Center	USA	19	1040	54.7
5	Indiana University	USA	16	719	44.9
6	Tohoku University	Japan	15	1031	68.7
7	Massachusetts General Hospital	USA	15	856	57.1
8	Kobe University	Japan	15	233	15.5
9	Sungkyunkwan University	South Korea	14	207	14.8
10	Hôpital Beaujon	France	13	957	73.6

IPMN = intraductal papillary mucinous neoplasms.

**Table 3:** Corresponding authors with 7 or more articles in the study on IPMN.

Rank	Corresponding author	Country	Number of studies	Total citations	Average citation
1	Tanaka, M	Japan	26	1143	44.0
2	Jang, JY	South Korea	13	318	24.5
3	Schmidt, CM	USA	12	666	55.5
4	Furukawa, T	Japan	11	964	87.6
5	Fernandez-del Castillo, C	USA	8	329	41.1
6	Nakagohri, T	Japan	8	180	22.5
7	Masuda, A	Japan	8	34	4.3
8	Yamaue, H	Japan	7	342	48.9

IPMN = intraductal papillary mucinous neoplasms.

### 3.2. Publication journals and citations

Journals publishing more than 20 articles in the study on IPMN were *Pancreas, Pancreatology, Annals of Surgery, Hepato-gastroenterology, Surgery*, and *Journal of Gastrointestinal Surgery. Pancreas* published the highest number of articles and subsequently had the highest number of citations (n = 100, citations = 2533) on IPMN, followed by *Pancreatology* (n = 48, citations = 872), and *Annals of Surgery* (n = 38, citations = 3789) (Table 4). Interestingly, the paper with the highest number of citations and the number of citations among articles is the same, the highest journal publishing and most cited IPMN article was *Annals of Surgery* (Impact factor = 12.969, citations = 3789). The *Hepato-gastroenterology* has been discontinued as of 2015. Thus, the impact factor of this journal is from 2015.

**Table 4:** Journals publishing more 20 articles in the study on IPMN.

Rank	Journal title	Impact factor 2020	Number of studies	Total citations
1	*Pancreas*	3.327	100	2533
2	*Pancreatology*	3.996	48	872
3	*Annals of Surgery*	12.969	38	3789
4	*Hepato-gastroenterology* *	0.792*	32	629
5	*Surgery*	3.982	28	900
6	*Journal of Gastrointestinal Surgery*	3.452	21	707

IPMN = intraductal papillary mucinous neoplasms.

* *Hepato-gastroenterology* has been discontinued as of 2015. Thus, the impact factor of this journal is from 2015’s.

### 3.3. Research focus, trend, and keyword analysis

**Figure 3.**
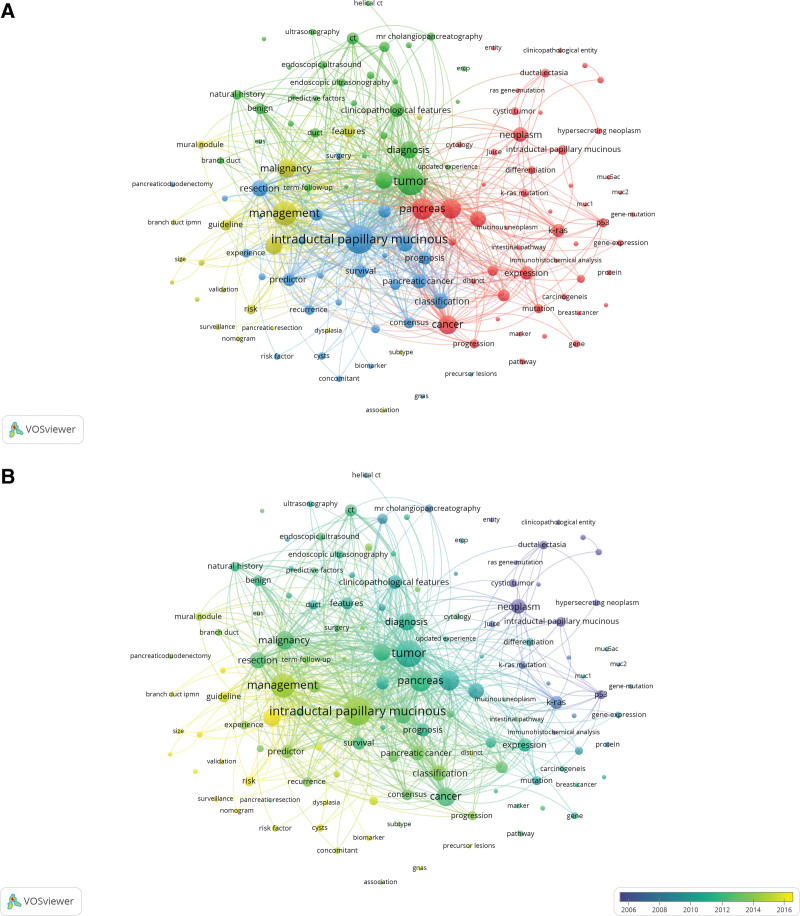
(A) The network visualization of keywords co-occurrence with frequency more 10 times. Circles with the same color belong to the same cluster. (B) The overlay visualization map of keywords based on the time of appearance. While the earliest nodes were painted with purple, the most recent ones were colored with yellow.

According to the topic of articles, the articles on basic research (n = 201) accounted for the largest portion, followed by malignant IPMN (n = 183) and disease overview including diagnosis (n = 171) (Figure S4, Supplemental Digital Content, http://links.lww.com/MD/I817). Publications on basic research and malignant IPMN showed a tendency to increase gradually compared to other topics. To explore the research hotspots in the field of Artificial intelligence on cancer research, VOS viewer was used for bibliometric keywords network analysis. Among 2053 keywords, 132 keywords that appeared more than 10 times were selected for cluster analysis, and they were grouped into 4 clusters (Fig. 3A). The red cluster mainly consisted of keywords related to basic research, in which the most common keywords were pancreas (228 times), carcinoma (199 times), and cancer (180 times) (Table S1, Supplemental Digital Content, http://links.lww.com/MD/I818). The green cluster mainly consisted of keywords related to disease overview of IPMN, in which the most common keywords were tumor (365 times), diagnosis (150 times), and cystic neoplasm (139 times). The blue cluster mainly consisted of keywords related to management and prognosis, in which the most common keywords were intraductal papillary mucinous neoplasm (398 times), IPMN (131 times), and resection (130 times). At last, the yellow cluster mainly consisted of keywords related to malignant IPMN, in which the most common keywords were management (297 times), and malignancy (171 times), and international consensus guideline (152 times).

Overlay visualization demonstrates the network map of the trend of topics according to the keywords (Fig. 3B). In the past, studies focused on basic research or disease overview were published, but recently, studies focusing on management or prognosis tend to be published. Articles on histological subtypes and EUS have increased in the last decade globally. Many studies on BD-IPMN have been published recently. In genetic mutations, many studies related to Kirsten-ras (KRAS), MUC (MUC1, MUC2 and MUC5AC), and p53 mutations was reported in the past. Recent research on GNAS mutations have been reported more frequently in IPMN.

## 4. Discussion

IPMN was first reported in Japan by Ohashi et al in 1982, and IPMN was reported as a rare case until the mid-1990s. However, the detection rate of asymptomatic pancreatic cystic lesions including IPMN is rapidly increasing in line with the popularization and the development of imaging modalities such as abdominal ultrasound, computed tomography, and magnetic resonance imaging.^[14]^ The first Sendai guidelines were published in 2006 that suggested the treatment policy for IPMN and follow-up monitoring intervals. Recently, subsequent Fukuoka guidelines and its revised guidelines were published in 2012 and 2017 each.^[5]^ Lately, the American Gastroenterological Association guideline published in 2015.^[15]^ A bibliometric analysis with keywords network analysis was performed in this study to determine the most cited and influential studies in the field of IPMN with the aim of systematically describing the focus and research trends of IPMN.

Because the incidence of pancreatic cystic lesion was low (2.6–15%), the number of articles is relatively small compared to other diseases.^[16]^ The reason may be the search strategy used in this study. To eliminate the heterogeneity of the study, we searched “IPMN” OR “intraductal papillary mucinous neoplasm” among the articles for the past 30 years. In previously reported bibliometric analysis of pancreatic cystic neoplasms, all pancreatic cystic lesions including solid pseudopapillary neoplasm, mucinous cystic neoplasm, and pancreatic pseudocyst were searched.^[9,10]^ Unlike other previously reported bibliometric analysis of IPMN, we excluded studies that included pancreatic cystic neoplasm or simple pancreas cyst.^[9]^

The rate of increase in the number of articles increased markedly until 2010, but it has been somewhat stagnant in recent years. To find the answer, we need to pay attention to the time when 2 guidelines (Sendai, Fukuoka) with revised version that suggested the treatment policy for IPMN, and follow-up monitoring intervals were established. As consensus was reached in the diagnosis, treatment, and follow-up period of IPMN, the direction and purpose of the study were established based on this. Since malignant transformation is observed during the long-term observation period, more studies with a longer follow-up period should be performed.

Interestingly, compared to other pancreatic disease studies, many studies have been conducted in Japan and Korea. In this study, Japan published the most articles, and Kyushu University was the institution that conducted the most extensive research and papers among the top 10 institutions with highest published articles in IPMN research. Tanaka, M published the most articles in the study on IPMN. Seoul National University published the second largest number of papers. Jang, JY published the second largest number of articles in the study on IPMN. These findings might be related to prevalence of the IPMN as it is known that IPMN is more common in the Asian ethnic groups.^[17]^

Unlike pancreatic cancer, it was published more in pancreas specific journals (*Pancreas, Pancreatology, Annals of Surgery, Hepato-gastroenterology, Surgery*, and *Journal of Gastrointestinal Surgery*) than in journals with a very high impact factor such as *New England Journal of Medicine, Science, Nature*, and *Lancet*.^[18]^ In comparing the previously reported bibliometric analysis of pancreatic cystic neoplasms, we found a similar pattern though the result was not completely consistent with our findings.^[10]^ The first reason was due to the low prevalence of the IPMN and the low interest of researchers. The second reason was majority of research on IPMN were retrospective cohort studies.

Using bibliometric keywords network analysis, we found the current research focus and trend of IPMN. While early IPMN research focused on basic science related to malignant transformation and diagnosis, recent studies focused on treatment methods and prognosis after treatment. These global changes in the approach to IPMN treatment and surveillance protocols impacts on current management strategies. Recent advances in the histological subtype analysis of IPMN allowed us to classify the IPMN into 4 groups. And this identification of subtype gives a greater understanding of the malignant transformation potential of IPMN. Novel diagnostic modality, endoscopic ultrasound-guided fine-needle aspiration/biopsy now can be popularly used for cytology and cystic fluid analysis. GNAS mutations have been reported more frequently in IPMN than KRAS, Mucins (MUC1, MUC2 and MUC5AC), and p53 mutations. Mutation in KRAS and MUC was common but not strongly associated with IPMN histologic progression.^[19]^ The malignant transformation of IPMN to an invasive carcinoma depends on the mutations of both P53 and P16.^[20]^ A previous study reported that mutations in GNAS at codon 201 had recently been identified as a hallmark mutation of IPMN.^[21]^ Some reports revealed that GNAS mutation was significantly related to high-grade dysplasia, while others reported that wild-type GNAS was significantly associated with adenocarcinoma.^[21,22]^

As with other bibliometric analyses, this study has some potential limitations. First, citation analysis in this study was based on Web of Science. Some articles indexed by other databases, such as Scopus and Google might be missed. Second, several biases may exist including the language bias, institutional bias, self-citation bias and powerful person bias. Third, older articles are likely to have more citations. Citation rate index was adopted to overcome the impact of this limitation. Finally, only corresponding authors and the institutions were considered in the analysis; thus, the contributions of all other authors were not fully included.

## 5. Conclusion

To our knowledge, this is the first bibliometric study to identify the most influential articles and institution in IPMN research over the past 30 years. Plus, this study analyzed global research trends in IPMN over the past 30 years and provided insight into the features and research hotspots of the articles in IPMN research. Thus, this study provides a new insight and perspective on the trends of IPMN research. Our knowledge of growing interest and trends of research in IPMN is essential for deeper understanding and optimal management of IPMN.

## Author contributions

**Conceptualization:** Ji Woong Hwang.

**Data curation:** Ji Woong Hwang.

**Formal analysis:** Jae Keun Park.

**Visualization:** Ji Woong Hwang.

**Writing – original draft:** Jae Keun Park.

**Writing – review & editing:** Jae Keun Park, Ji Woong Hwang.

## Supplementary Material











## References

[1] Kawakubo K Tada M Isayama H. Incidence of extrapancreatic malignancies in patients with intraductal papillary mucinous neoplasms of the pancreas. Gut. 2011;60:1249–53. 21398676 10.1136/gut.2010.227306

[2] Kleeff J Korc M Apte M. Pancreatic cancer. Nat Rev Dis Primers. 2016;2:1–22. 10.1038/nrdp.2016.22 27158978

[3] Tanaka M Chari S Adsay V. International association of pancreatology: international consensus guidelines for management of intraductal papillary mucinous neoplasms and mucinous cystic neoplasms of the pancreas. Pancreatology. 2006;6:17–32. 16327281 10.1159/000090023

[4] Assarzadegan N Babanianmansour S Shi J. Updates in the diagnosis of intraductal neoplasms of the pancreas [in English]. Front Physiol. 2022;13:856803. 35309060 10.3389/fphys.2022.856803 PMC8931033

[5] Tanaka M Fernández-del Castillo C Kamisawa T. Revisions of international consensus Fukuoka guidelines for the management of IPMN of the pancreas. Pancreatology. 2017;17:738–53. 28735806 10.1016/j.pan.2017.07.007

[6] Keane MG Afghani E. A review of the diagnosis and management of premalignant pancreatic cystic lesions. J Clin Med. 2021;10:1284. 33808853 10.3390/jcm10061284 PMC8003622

[7] Furukawa T. Subtyping of IPMN. Methods Mol Biol. 2019;1882:1–9. 30378039 10.1007/978-1-4939-8879-2_1

[8] Luukkonen T. Invited review article: bibliometrics and evaluation of research performance. Ann Med. 1990;22:145–50. 2393549 10.3109/07853899009147259

[9] Hughes D Hughes I Powell A. Intraductal papillary mucinous neoplasm’s 100 most significant manuscripts: a bibliometric analysis. Int J Hepatobiliary Pancreat Dis. 2018;8:100076Z04DH2018.

[10] Cui M Hu Y You L. A bibliometric study on pancreatic cystic disease research. J Pancreatol. 2019;2:43–7.

[11] Moher D Shamseer L Clarke M. Preferred reporting items for systematic review and meta-analysis protocols (PRISMA-P) 2015 statement [in English]. Syst Rev. 2015;4:1. 25554246 10.1186/2046-4053-4-1 PMC4320440

[12] van Eck NJ Waltman L. Software survey: VOS viewer, a computer program for bibliometric mapping [in English]. Scientometrics. 2010;84:523–38. 20585380 10.1007/s11192-009-0146-3 PMC2883932

[13] Shi S Gao Y Liu M. Top 100 most-cited articles on exosomes in the field of cancer: a bibliometric analysis and evidence mapping [in English]. Clin Exp Med. 2021;21:181–94. 32266495 10.1007/s10238-020-00624-5

[14] Brugge WR Lauwers GY Sahani D. Cystic neoplasms of the pancreas. N Engl J Med. 2004;351:1218–26. 15371579 10.1056/NEJMra031623

[15] Vege SS Ziring B Jain R. American gastroenterological association institute guideline on the diagnosis and management of asymptomatic neoplastic pancreatic cysts. Gastroenterology. 2015;148:819–22. 25805375 10.1053/j.gastro.2015.01.015

[16] Farrell JJ. Prevalence, diagnosis and management of pancreatic cystic neoplasms: current status and future directions. Gut Liver. 2015;9:571–89. 26343068 10.5009/gnl15063 PMC4562774

[17] Laffan TA Horton KM Klein AP. Prevalence of unsuspected pancreatic cysts on MDCT. AJR Am J Roentgenol. 2008;191:802–7. 18716113 10.2214/AJR.07.3340 PMC2692243

[18] Li Q Jiang Y. Top classic citations in pancreatic cancer research. World J Surg Oncol. 2016;14:1–8. 27899147 10.1186/s12957-016-1061-8 PMC5129593

[19] Nissim S Idos GE Wu B. Genetic markers of malignant transformation in intraductal papillary mucinous neoplasm of the pancreas: a meta-analysis. Pancreas. 2012;41:1195–205. 22750975 10.1097/MPA.0b013e3182580fb4 PMC4850028

[20] Moris D Damaskos C Spartalis E. Updates and critical evaluation on novel biomarkers for the malignant progression of intraductal papillary mucinous neoplasms of the pancreas. Anticancer Res. 2017;37:2185–94. 28476781 10.21873/anticanres.11553

[21] Wu J Matthaei H Maitra A. Recurrent GNAS mutations define an unexpected pathway for pancreatic cyst development. Sci Transl Med. 2011;3:92ra66. 10.1126/scitranslmed.3002543 PMC3160649 21775669

[22] Ideno N Ohtsuka T Matsunaga T. Clinical significance of GNAS mutation in intraductal papillary mucinous neoplasm of the pancreas with concomitant pancreatic ductal adenocarcinoma. Pancreas. 2015;44:311–20. 25479586 10.1097/MPA.0000000000000258

