# Supplementary figures and images for: Research progress and future directions on intraductal papillary mucinous neoplasm: A bibliometric and visualized analysis of over 30 years of research
Source: Medicine (Baltimore). 2023 Apr 14;102(15):e33568. doi: 10.1097/MD.0000000000033568 (PMC10101262; doi:10.1097/MD.0000000000033568)

**Figure S1.** Annual publication on intraductal papillary mucinous neoplasm

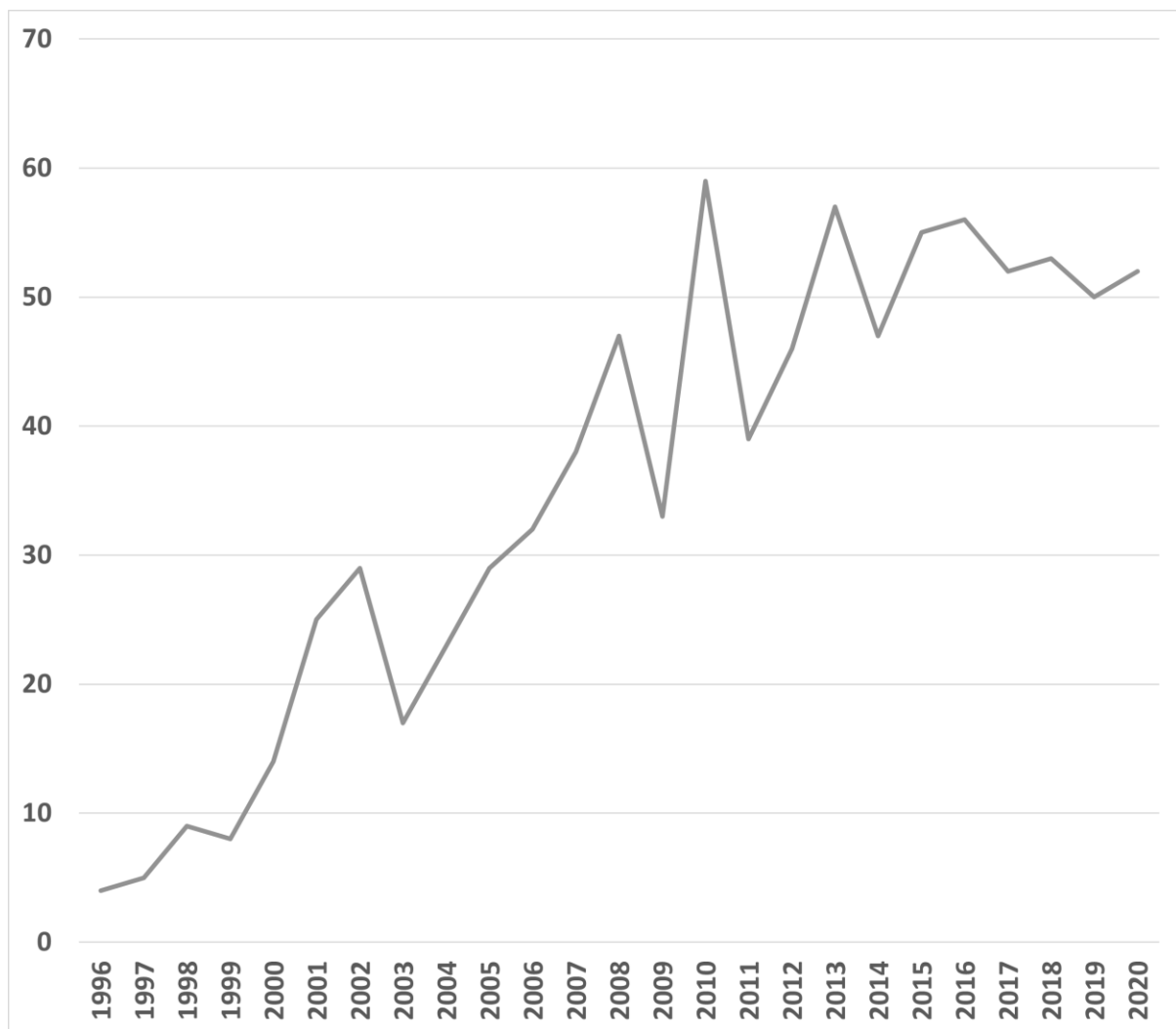

Supplement: Supplementary file 1 [file medi-102-e33568-s001.pdf]

**Figure S3.** Status of IPMN-related multicenter studies in each nation

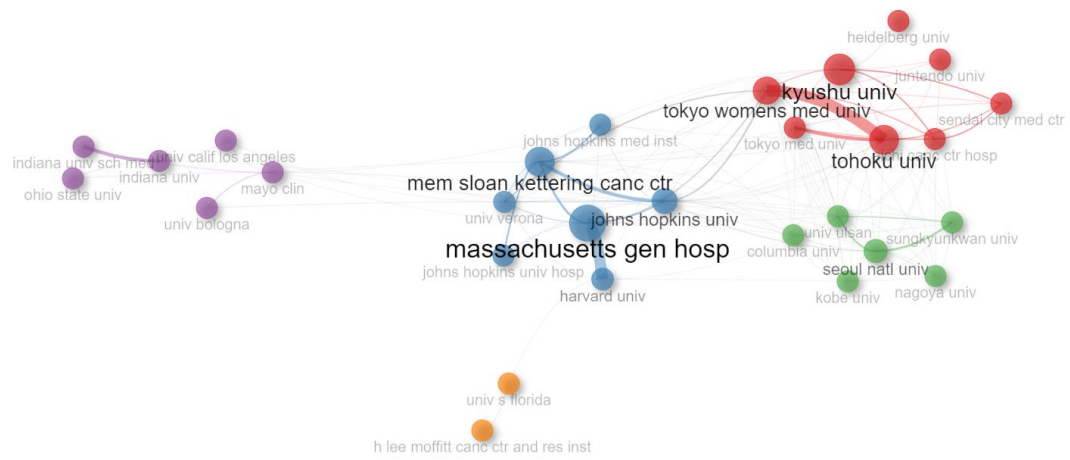

Supplement: Supplementary file 3 [file medi-102-e33568-s003.pdf]
