# Supplementary material for: Research progress and future directions on intraductal papillary mucinous neoplasm: A bibliometric and visualized analysis of over 30 years of research
Source: Medicine (Baltimore). 2023 Apr 14;102(15):e33568. doi: 10.1097/MD.0000000000033568 (PMC10101262; doi:10.1097/MD.0000000000033568)

Figure S2. Intraductal papillary mucinous neoplasm research status of multicenter studies by nation

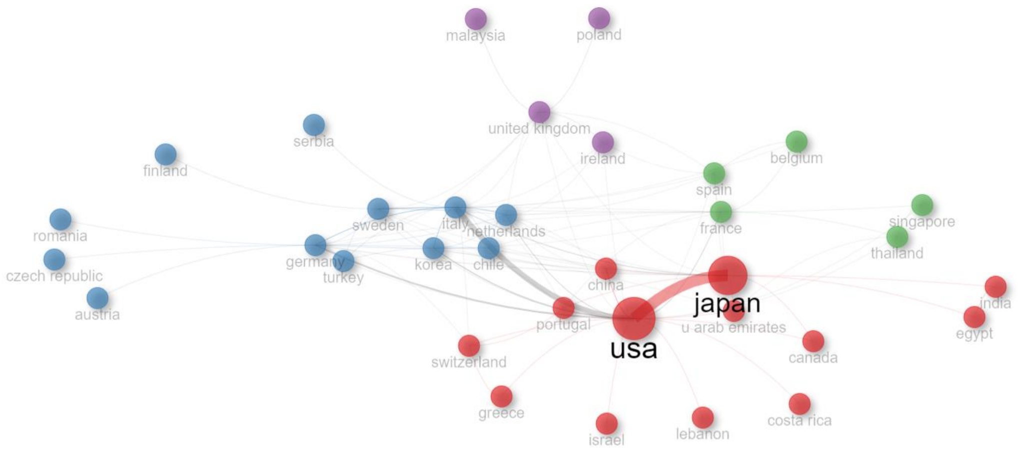

Supplement: Supplementary file 2 [file medi-102-e33568-s002.pdf]
