# Supplementary material for: Research progress and future directions on intraductal papillary mucinous neoplasm: A bibliometric and visualized analysis of over 30 years of research
Source: Medicine (Baltimore). 2023 Apr 14;102(15):e33568. doi: 10.1097/MD.0000000000033568 (PMC10101262; doi:10.1097/MD.0000000000033568)
Supplement: Supplementary file 5 [file medi-102-e33568-s005.pdf]

Table S1. List of keywords of all articles generated by VOSviewer.

Keyword	Cluster	Links	Total link strength	Occurrences	Average published year
pancreas	1	129	1689	228	2011.6842
carcinoma	1	128	1440	199	2010.0854
cancer	1	120	1177	180	2012.2167
adenocarcinoma	1	118	878	123	2009.5122
neoplasm	1	108	607	105	2004.6476
expression	1	100	602	89	2011.0674
k-ras	1	96	548	82	2007.3659
ductal adenocarcinoma	1	98	515	73	2012.726
intraepithelial neoplasia	1	94	420	56	2011.0893
intraductal papillary mucinous tumor	1	87	346	51	2003.5686
p53	1	74	327	46	2004.4783
progression	1	86	320	46	2013.7174
mutation	1	75	296	45	2009.7333
ductal ectasia	1	64	268	41	2001.4634
immunohistochemistry	1	72	212	33	2008.9394
cystic tumor	1	80	231	32	2004.375
differentiation	1	77	247	32	2009.4688
invasive carcinoma	1	87	270	31	2012.6129
k-ras mutation	1	83	229	31	2006.7742
gene	1	51	163	27	2010.5185
hypersecreting neoplasm	1	49	161	24	2001.2083
gene-expression	1	48	147	23	2008.8696
juice	1	63	172	22	2007.2273
cystadenocarcinoma	1	45	155	21	2001.6667
carcinogeneis	1	55	140	20	2010.95
cytology	1	61	156	20	2012.25
pathway	1	56	132	19	2012.3158
cystadenoma	1	43	122	17	2002.9412
protein	1	47	112	17	2007.9412
distinct	1	57	123	15	2015.3333
breast-cancer	1	41	67	12	2011.1667
clinicopathological entity	1	42	92	12	2002.6667
colorectal-cancer	1	42	69	12	2012.1667
gene-mutation	1	46	99	12	2007.6667
marker	1	39	64	12	2011.75
noncystic carcinoma	1	48	101	12	2007.75

ras gene-mutation	1	48	96	12	2004.5833
cells	1	39	68	11	2009.9091
intestinal pathway	1	48	95	11	2013.2727
muc2	1	40	79	11	2008.8182
pancreatic intraepithelial neoplasia	1	49	93	11	2011.7273
pancreatic juice	1	56	96	11	2010.3636
pancreatic tumor	1	50	87	11	2005.7273
point mutation	1	43	83	11	2007.3636
entity	1	37	68	10	2003.5
immunohistochemical analysis	1	38	68	10	2005.7
muc1	1	43	92	10	2010.3
muc5ac	1	42	83	10	2008.6
mucinous neoplasm	1	46	74	10	2012.2
pancreatic carcinoma	1	43	68	10	2007.6
tumor	2	131	2515	365	2010.6274
diagnosis	2	126	1113	150	2010.98
cystic neoplasm	2	124	1074	139	2011.6403
clinicopathological features	2	104	803	94	2009.5319
ct	2	87	554	70	2012.0429
benign	2	89	504	60	2011.5833
differential-diagnosis	2	91	452	55	2008.6909
natural history	2	78	436	51	2011.8235
duct	2	81	345	45	2009.8444
mr cholangiopancreatography	2	76	283	36	2007.8056
endoscopic ultrasonography	2	81	260	33	2011.0909
endoscopic ultrasound	2	79	296	32	2012.1875
fine-needle-aspiration	2	77	243	29	2013.7241
term-follow-up	2	85	254	29	2013.4483
pancreatic neoplasm	2	81	207	26	2009.6538
branch duct	2	64	234	25	2013.64
ultrasonography	2	56	159	22	2009.7273
cystic lesions	2	58	136	20	2011.3
peroral pancreatoscopy	2	58	157	20	2010.8
surgical-management	2	63	149	19	2009.7368
predictive factors	2	61	161	18	2012.3333
helical ct	2	52	139	17	2008.4118
ercp	2	59	121	16	2009.625
extrapancreatic neoplasm	2	54	126	16	2011.375
branch duct type	2	58	134	15	2011.0667

updated experience	2	55	113	15	2010.4
eus	2	53	116	14	2013.3571
juice cytology	2	43	99	13	2013.7692
head resection	2	44	71	12	2009.1667
clinical-characteristics	2	41	80	11	2013.6364
positron-emission-tomography	2	42	80	11	2012.7273
in-situ	2	45	80	10	2008.7
intraductal papillary mucinous neoplasm	3	131	2729	398	2014
ipmn	3	123	936	131	2012.5115
resection	3	107	979	130	2012.3385
classification	3	117	970	127	2013.4803
pancreatic cancer	3	112	662	99	2013.1919
predictor	3	101	707	87	2013.8276
prognosis	3	104	669	87	2011.2874
survival	3	101	629	85	2012.0471
surgical resection	3	100	595	76	2010.3684
follow-up	3	106	668	75	2013.68
consensus	3	91	481	62	2012.9032
lesions	3	92	381	55	2013.2545
recurrence	3	76	388	48	2013.625
experience	3	75	310	38	2013.7895
pancreatic ductal adenocarcinoma	3	80	295	37	2015.1892
outcome	3	68	258	34	2012.8235
cysts	3	71	196	28	2015.3214
pancreatectomy	3	60	183	25	2013.32
surgery	3	70	166	22	2012.5909
concomitant	3	57	185	21	2014.2857
prevalence	3	54	158	21	2014.8095
risk factor	3	59	155	21	2015.1429
consensus guideline	3	63	142	20	2016.8
remnant pancreas	3	65	165	19	2016.5789
biomarker	3	41	96	15	2016.8667
ductal carcinoma	3	61	130	15	2011.2
pancreaticoduodenectomy	3	42	89	15	2013.8
precursor lesions	3	47	90	11	2015
gnas	3	34	64	10	2017.7
total pancreatectomy	3	43	73	10	2013.9
management	4	123	2219	297	2014.2761
malignancy	4	121	1454	171	2012.3801

international consensus guideline	4	112	1210	152	2016.1645
features	4	92	496	74	2010.5811
guideline	4	88	455	57	2015.2281
risk	4	79	383	51	2015.9412
mural nodule	4	77	322	36	2014.6111
carcinoembryonic antigen	4	53	169	19	2016.8421
mri	4	53	140	17	2013.3529
size	4	53	146	16	2016.625
surveillance	4	58	136	16	2016.8125
validation	4	48	107	15	2018.2667
branch duct ipmn	4	46	131	14	2016.1429
metaanalysis	4	49	96	13	2016.3077
nomogram	4	50	123	13	2018.2308
dysplasia	4	54	111	12	2016.25
carbohydrate antigen 19-9	4	38	105	11	2017.3636
subtype	4	47	89	11	2013.6364
association	4	34	60	10	2013.4
pancreatic resection	4	47	94	10	2012.6
